# Stress-Dependent Particle Interactions of Magnesium Aluminometasilicates as Their Performance Factor in Powder Flow and Compaction Applications

**DOI:** 10.3390/ma14040900

**Published:** 2021-02-14

**Authors:** Pavlína Komínová, Lukáš Kulaviak, Petr Zámostný

**Affiliations:** 1 Department of Organic Technology, University of Chemistry and Technology Prague, Technická 5, 166 28 Prague, Czech Republic; 2 Department of Multiphase Reactors, Institute of Chemical Process Fundamentals, Czech Academy of Sciences, Rozvojová 135, 165 02 Prague, Czech Republic; kulaviak@icpf.cas.cz

**Keywords:** glidant, particle interactions, threshold behavior, magnesium aluminometasilicates, formulation development

## Abstract

In the pharmaceutical industry, silicates are commonly used excipients with different application possibilities. They are especially utilized as glidants in low concentrations, but they can be used in high concentrations as porous carriers and coating materials in oral solid drug delivery systems. The desirable formulations of such systems must exhibit good powder flow but also good compactibility, which brings opposing requirements on inter-particle interactions. Since magnesium aluminometasilicates (MAS) are known for their interesting flow behavior reported as “negative cohesivity” yet they can be used as binders for tablet compression, the objective of this experimental study was to investigate their particle interactions within a broad range of mechanical stress from several kPa to hundreds of MPa. Magnesium aluminometasilicate (Neusilin^®^ US2 and Neusilin^®^ S2)-microcrystalline cellulose (Avicel^®^ PH102) physical powder mixtures with varying silicate concentrations were prepared and examined during their exposure to different pressures using powder rheology and compaction analysis. The results revealed that MAS particles retain their repulsive character and small contact surface area under normal conditions. If threshold pressure is applied, the destruction of MAS particles and formation of new surfaces leading to particle interactions are observed. The ability of MAS particles to form interactions intensifies with increasing pressure and their amount in a mixture. This “function switching” makes MAS suitable for use as multifunctional excipients since they can act as a glidant or a binder depending on the applied pressure.

## 1. Introduction

Silicates are ordinarily used as inactive materials in pharmaceutical industry. Different articles have been published in scientific literature dealing with the study of silicates with respect to their possible application purpose. A variety of silicate materials has been explored. In addition to the application in the pharmaceutical industry discussed below, silicates are widely used in a number of other branches of science and industry, such as adsorption of hazardous pollutants, catalysis, enzyme immobilization, optics industry, and others [1,2,3,4,5,6].

Silicates are predominantly valued for their beneficial physico-chemical properties. Regular silicates are well known for their great specific surface area and small particles in range of nanometers [7]. The small particle size makes them suitable for application as glidants [8,9]. Their primary particles are strongly adsorbed on the surface of bigger host particles during blending [10,11]. This leads to improved powder flow properties by either decreasing the number of attractive forces between host particles or the diminution of particle interlocking [12,13,14]. A number of extensive and thorough studies dealing with the effect of silicates on improvement in flow properties of several pharmaceutical excipients have been performed. For example, Davé et al. studied the effect of silica adsorbed on the host particle surface with considerations of processing techniques used for dry host particle coatings [15,16,17,18]. Literature also mentions a possibility of reduction of adsorbed moisture accompanied by the decrease of interparticle cohesion and adhesion as another way of flow improvement [19]. Silicate materials that are most frequently used as glidants are colloidal silica (e.g., Aerosil^®^ (Evonik Industries AG, North Rhine-Westphalia, Essen Germany), Cab-o-sil^®^ (Cabot Corporation, Boston. MA, USA), Sipernat^®^ (Evonik Industries AG, North Rhine-Westphalia, Essen, Germany), Syloid^®^ (W.R. Grace & Co., Columbia, MD, USA) or magnesium silicate (e.g., talc) [9,17,20,21].

Moreover, silicate materials are also used as carriers. Due to the large specific surface area, they are well suited for the adsorption of solid as well as liquid drugs or preparation of liquisolid systems (LS) and self-emulsifying drug delivery systems (SEDDS). One of the most favorable carrier materials is Silica Aerogel [22,23]. Calcium silicate, colloidal silica (Aerosil^®^), kaolin (Lion^®^ (CB Minerals, Mamaroneck, NY, USA)), Sim^®^ 90 (Universal Preserv-A-Chem Inc., Somerset, UK, )), ordered mesoporous silicate (OMS) (SBA-15, MCM-41, TUD-1), or silica gel (Syloid^®^, Sylysia^®^ (Fuji Silysia Chemical Ltd., Kasugai Aichi, Japan)) are also used [24,25,26,27]. Some silicates, especially those with small particle size, may find application as coating materials. Colloidal silica (Aerosil^®^, Cab-O-Sil^®^) belongs among ones exhibiting this characteristic [28,29]. Amorphous silica gel (Syloid^®^, Sylysia^®^), granulated silicon dioxide (Aeroperl^®^ (Evonik Industries AG, North Rhine-Westphalia, Essen, Germany)), Silica Aerogel, calcium silicate (Florite^®^ (Tomita Pharmaceutical Co., Ltd., Tokushima, Japan)), and ordered mesoporous silicate can be also taken into consideration for application [30,31,32].

Other silicate materials with very extraordinary and suitable properties are magnesium aluminometasilicates (MAS). The most prominent representative is Neusilin^®^. It is an amorphous and a synthetic type of MAS available either in a fine powder or a granule form. The most known and used grades are S1, S2, UFL2, and US2. Neusilin^®^ is widely used for the improvement of the quality of tablets (in terms of compaction and disintegration properties), powders, granules, and capsules. It can be used in direct compression and also in wet granulation. It exhibits an extremely large specific surface area and high porosity. Neusilin^®^ is also superior in compressibility and compactibility, which allows to create hard tablets at low compression forces. The high porosity and large specific surface area give Neusilin^®^ significant adsorption capacity [33]. With respect to this, Neusilin^®^ is an excellent carrier for solid drug delivery systems and hot-melt extrusion [34,35,36,37,38,39]. Moreover, it is known for its ability to improve powder flow properties [33,40,41,42]. Neusilin^®^ also evinces a predisposition to the improvement of stability and bioavailability of active pharmaceuticals ingredients [26,43,44,45,46,47].

In case of silicate application as a glidant, the usual concentration in a formulation is up to 1%. Currently, available literature concerning this topic is mainly focused on finding new glidants, optimal process conditions leading to the highest glidant effectiveness, or further understanding of glidant mechanism of action [48,49,50,51,52,53,54].

In comparison to the application as glidants, the use of silicates as carrier materials in solid drug delivery systems requires higher amounts. As tablets represent common solid dosage forms, the focused literature discusses mainly the impact of composition of studied drug delivery systems and type of used silicates on tabletability and physical parameters of prepared tablets such as tensile strength or dissolution behavior [19,26,29,38].

It is obvious some silicates find their utilization in both the improvement of flow properties and the preparation of solid drug delivery systems. However, a literature survey has revealed mainly the availability of studies concerning the application of silicates either as glidants or carriers (coating materials).

The main goal of this study was to provide initial screening outlining the relation between mixture composition (amount of silicate), applied load, and final silicate behavior. Obtained findings may result in better understanding of conditions under which silicates incline to their glidant or carrier usage. For the purpose of the study, model binary powder mixtures consisting of microcrystalline cellulose (MCC, Avicel^®^ PH102; FMC Bio Polymers, Philadelphia, PA, USA) and various concentrations of MAS were prepared and analyzed across a broad range of applied load. Neusilin^®^ US2 and Neusilin^®^ S2 (Fuji Chemical Industries Co., Toyama, Japan) were employed as MAS representatives in the study.

Neusilin^®^ US2 and Neusilin^®^ S2 themselves show excellent flow properties [55,56]. However, their ability to improve flow properties of other excipients has not been well examined so far, e.g., in comparison with the grade Neusilin^®^ UFL2 (Fuji Chemical Industries Co., Toyama, Japan) known for its glidant activity [33,40]. On the other hand, complementary flow parameters, studied in scientific literature focused on different topics, suggest their possible utilization as a glidant [29,57] where their granular character could be beneficial compared to the traditional nanometer-sized glidants. Taking into consideration the main application of Neusilin^®^ US2 and Neusilin^®^ S2 as a carrier, a tablet hardener, or an enhancer of amorphous active pharmaceutical ingredient stability, further understanding of their possible multifunctional role in pharmaceutical formulations could be highly beneficial. For this reason, the above-mentioned grades of MAS were selected for evaluation.

## 2. Materials and Methods

### 2.1. Materials

The materials used were microcrystalline cellulose (MCC, Avicel^®^ PH102; FMC Bio Polymers, Philadelphia, PA, USA) and magnesium aluminometasilicate (Neusilin^®^ US2, Neusilin^®^ S2; Fuji Chemical Industries Co., Toyama, Japan).

Particle size was determined for each material using the laser diffraction particle size analyzer (Mastersizer 3000; Malvern Instruments, Worcestershire, Malvern, UK) equipped with a wet dispersion unit.

### 2.2. Preparation of Physical Powder Mixtures

In the case of microcrystalline cellulose, the initial material was sieved using vibratory sieve shaker model AS 2000 Basic (Retsch, Germany, North Rhine-Westphalia, Haan) to obtain two size fractions 100–150 µm and 150–250 µm. Neusilin^®^ US2 and Neusilin^®^ S2 were used in the study without any adjustment.

Fourteen binary mixtures containing selected particle fraction of Avicel^®^ PH102 (FMC Bio Polymers, Philadelphia, PA, USA) and various concentration of Neusilin^®^ US2 or Neusilin^®^ S2 grades were prepared. The ingredients were accurately weighed, transferred into plastic vessel, and then mixed together. The mixing was performed using the Turbula tumbling blender T2F (W. A. Bachofen, Basel, Switzerland) at 50 rpm for a period of 10 min. The concentrations of respective Neusilin^®^ grade were set by weight given the total weight of formulations. The prepared samples were stored in sealed containers to avoid the effect of atmospheric moisture. The list of prepared samples is reviewed in Table 1.

The mixture compositions and selected preparation procedures are patterned on previous studies carried out by the authors [52,58]. These studies are oriented on the characterization of colloidal silicone dioxide (Aerosil^®^ 200; Evonik Industries AG, North Rhine-Westphalia, Essen, Germany) as a traditional glidant and in this study presented MAS (Neusilin^®^ US2 and Neusilin^®^ S2) as a new potential type of glidant with a different mechanism of action. Authors are aware of the fact that connection of this article with their previous works brings some limitations in the case of possible experimental design setting and several categorical factors such as particle size, glidant content, or particle shape are fully or partially out of considerations. However, this comparison design of the article allows to obtain directly comparable results and get a more complex view on the topic of glidant application in solid dosage forms. Since the concept set in the previous works was retained in this study, revolutions per minute were set at value of 50. Mixing time of 10 min was chosen as suitable for mixture comparison in this study because the authors in their previous work focused on MAS revealed that total energy (*TE*) obtained during repeated energy measurements of one sample via FT4 powder rheometer exhibited stable values. With respect to the fact that mixture preparation was carried out using effective 3D blending and powder motion in rheometer is very similar to intensive stirring, these findings indicate that powders reach their equilibrium homogeneity at this mixing time. This statement will be also further confirmed by discussion of images in Section 3.2. The lower amounts of MAS in concentrations of 1 and 5 percent were chosen for possible comparison with traditional glidants. Mixtures containing 10 and 25 percent of MAS were prepared to identify a different mechanism of action and binder ability of MAS, respectively. Microcrystalline cellulose was selected because it is a widely used material in several industry sectors. In the case of pharmaceutical industry, Avicel^®^ PH102 belongs to the most commonly used excipients in tablet direct compression. Since the mechanism of action of traditional glidants is dependent on the particle size of host particles, two fractions of microcrystalline cellulose were used in previous studies. In order to maintain the established concept and make the comparison of results feasible, the fractions were applied in this study as well.

### 2.3. Quantification of Particle Interaction

For the purpose of characterizing particle interactions with respect to powder consolidation state, two methods were involved in this research—shear cell methodology and powder compaction study for low (marginal) and high applied pressures, respectively.

#### 2.3.1. Analysis of Particle Interactions under Marginal or Mild Stress Condition

The shear cell test measures the shear strength of powder. That means it defines the shear stress needed to obtain the failure of powder (i.e., the powder particles start to move relative to one another) as a function of normal stress applied in the range of kPa. As defined by Schulze, this is usually performed for five levels of normal stress (e.g., 3, 4, 5, 6, and 7 kPa for normal stress of 9 kPa applied at so called pre-shear point). Five obtained data points are plotted in two-dimensional co-ordinate system (the normal stress values are plotted on an *x* axis and the corresponding shear stress values are plotted on a *y* axis). The line passing through these five points is called a yield locus and is the basis of parameters obtained during the shear test, such as the effective angle of internal friction (*Φ*_E_). A detailed description of the shear cell methodology can be found in available literature [59,60,61].

The powder’s shear properties of pure compounds as well as their binary mixtures at low pressures were studied using the FT4 powder rheometer (Freeman Technology Ltd., Tewkesbury, Gloucestershire, UK) with a 48 mm shear cell. Measurements were carried out in 50 mm × 85 mL split vessel.

The effective angle of internal friction served as the main parameter for determining particle interactions under low normal pressures. This angle is the measure of interparticle friction, and it indicates the ability of powder particles to flow against each other. A high value means more pronounced friction connected with the presence of interparticle interaction and less free-flowing powder. Angle values were defined for the pre-shear normal pressure *p* = 3, 9, 15 kPa.

#### 2.3.2. Analysis of Particle Interactions under Compaction Condition

During the powder compaction study, tablet formation (or tabletability) describes the ability of a powder material to be transformed into a specified strength compact through the creation of particle interactions under the effect of applied compaction pressure in a range of MPa. The obtained strength is usually evaluated via the tensile strength of tablets (*σ*_t_). In this study, GTP-1 compaction analyzer (Gamlen Tabletting, Beckenham, London, UK) was used for preparing compacts (tablets) and measuring compaction parameters. Mixture samples were compacted directly into tablets via a flat round punch with a diameter of 5 mm. Four terminal pressure values, *p* = 5, 50, 100 and 150 MPa, were applied. All compaction measurements were performed in the fixed load mode under force control. Displacement speed of a punch was set at 60 mm/min and compaction load was defined to obtain desired terminal pressures. After compression to the specified load, the punch immediately retracted by 1 mm. Tensile strength of the tablets was analyzed via tablet hardness tester Multitest 50 (Sotax, Switzerland, Basel-Landschaft, Aesch) and calculated using Equation (1):

(1) $$\sigma_{t}=\frac{2\cdot F}{\pi \cdot D\cdot h}$$

where σ_t_ is the tensile strength (Pa), *F* is the maximal diametrical crushing (fracture) force (N), *D* is the tablet diameter (m), and *h* is the tablet thickness (m) [62].

Obtained tensile strength was utilized as the parameter reflecting the presence and amount of particle interactions at higher applied pressures. In general, tablet tensile strength can be investigated as a function of compaction pressure but also as a function of composition. In this article, tensile strength is examined as a function of compaction pressure and composition of mixtures serves as a categorical factor. Data analysis using this statistical approach makes it possible to compare the results from the two applied measurement types (shear cell and compaction measurement). Moreover, the effect of compaction pressure on each glidant content can be evaluated as well.

### 2.4. Visual Observation

The mixtures/tablet cross-sections were observed using scanning electron microscope (SEM) images to monitor MAS particle interactions and facilitate understanding of the processes responsible for the bond interactions. The samples were investigated using TESCAN LYRA3GMU microscope (TESCAN ORSAY HOLDING a.s., Brno, Czech Republic) at accelerating voltage of 10 kV. Both secondary electron (SE) and backscattered electron (BSE) detection types were employed. Each mixture was coated with a thin layer of gold (Q150R ES; Quorum Technologies Ltd., Laughton, East Sussex, UK) before imaging to neutralize charging effects and increase an SE yield at final micrographs.

## 3. Results and Discussion

### 3.1. Powder Characterization

The obtained size distribution and shape characteristics of the excipients are listed in Table 2.

### 3.2. SEM/BSE Observation

For selected mixtures, images were taken using a scanning electron microscope to help better understand the processes involved in studied mixtures responsible for a Neusilin^®^ behavior. The scanning was done with the involvement of BSE detector which provides information about the relative value of mean atomic number as the brightness of the certain place in the image. Thus, the Neusilin^®^ particles are relatively brighter in the images compared to the microcrystalline cellulose ones.

**Figure 1:**
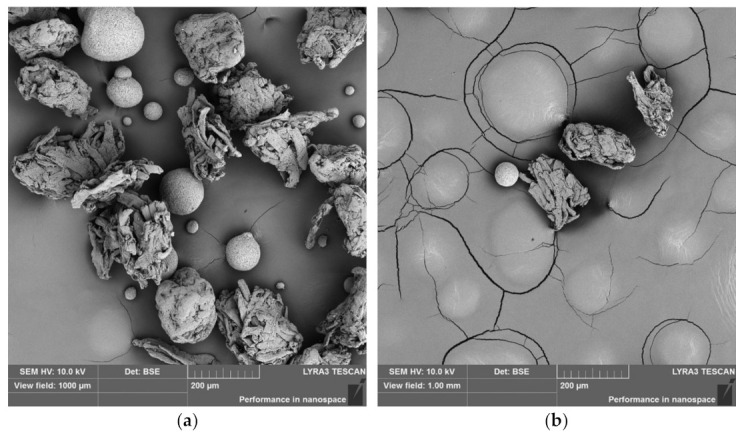
SEM/BSE (backscattered electron) image of microcrystalline cellulose mixture with 10% of Neusilin^®^ US2 (**a**) or 1% Neusilin^®^ S2 (**b**).

Figure 1a shows the image of powder mixture containing 10% of Neusilin^®^ US2. It is evident that Neusilin^®^ US2 particles (spherical ones) do not interact with one another nor with the microcrystalline cellulose particles. Thus, this image points out the repulsive behavior of Neusilin^®^ US2 particles under no or low-pressure conditions.

The visual observation of the second MAS confirms similar findings. In Figure 1b presenting the image of mixture with 1% Neusilin^®^ S2, the particles of Neusilin^®^ S2 also evince sphericity, bigger particle size, and lack of interaction forces. This highlights the similarity between the two studied MAS grades.

**Figure 2:**
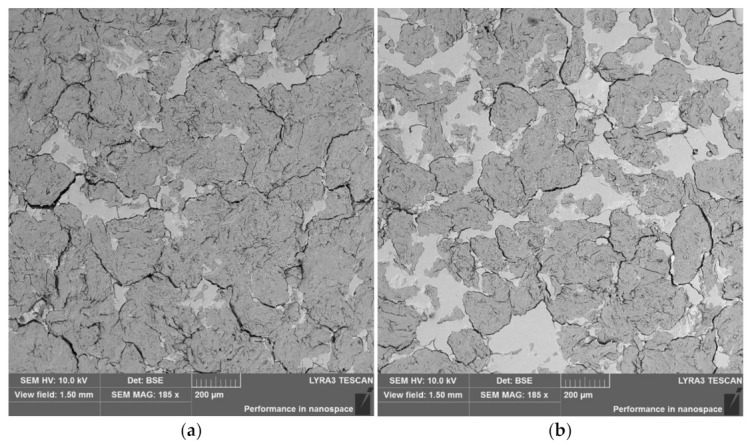
SEM/BSE images of cross-section of tablet compressed from mixture of microcrystalline cellulose and 5% (**a**) or 25% (**b**) of Neusilin^®^ US2.

In contrast, if the pressure is applied, the MAS particle interactions occur. It is obvious from the SEM/BSE images of internal cross-section of tablet prepared from the mixture with 5% and 25% of Neusilin^®^ US2 that are given in Figure 2. We can see Neusilin^®^ US2 spherical particles undergo significant destruction. This change in particle structure leads to increased surface area and an apparent switch from repulsive character to inter-particle bonding activity. The images also indicate MAS particle interconnections and their possible function as a reinforcement. On the side note, it is also important the solid phases are clearly distinguished and uniformly distributed in samples at scale of scrutiny in the order of millimeters, which confirms the uniformity of the starting mixture as mentioned in the experimental section.

### 3.3. Effect of Applied Pressure on Particle Interaction Creation

**Figure 3:**
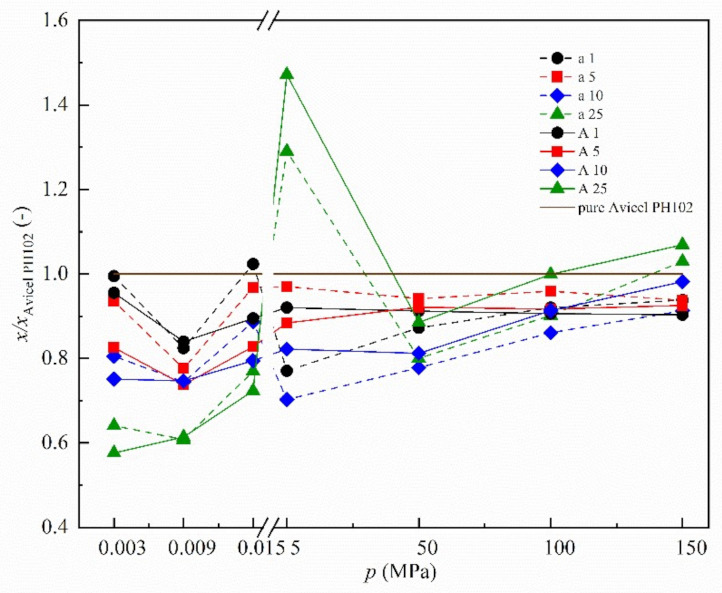
Effect of applied pressure on mixture containing Neusilin^®^ US2. Samples are identified by X-Y abbreviation where X = A-the coarse fraction of microcrystalline cellulose (—), X = a-the finer fraction of microcrystalline cellulose (- - -), Y = 1, 5, 10 and 25—1% (●), 5% (■), 10% (◆) and 25% (▲) of Neusilin^®^ US2. The *x/x*_Avicel PH102_ means the ratio of the value measured for the mixture to the value measured for pure microcrystalline cellulose. The *x* means the effective angle of internal friction for applied pressure up to 15 kPa and tensile strength for remaining applied pressures. (Standard deviation is not presented due to the graph readability).

**Figure 4:**
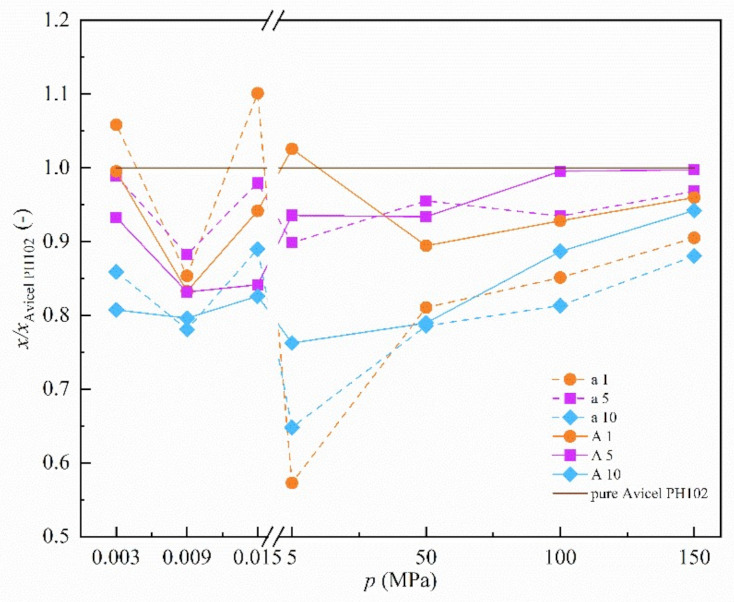
Effect of applied pressure on mixture containing Neusilin^®^ S2. Samples are identified by X-Y abbreviation where X = A-the coarse fraction of microcrystalline cellulose (—), X = a-the finer fraction of microcrystalline cellulose (- - -), Y = 1, 5 and 10—1% (●), 5% (■) and 10% (◆) of Neusilin^®^ S2. The *x/x*_Avicel PH102_ means the ratio of the value measured for mixture to the value measured for pure microcrystalline cellulose. The *x* means effective angle of internal friction for applied pressure up to 15 kPa and tensile strength for remaining applied pressures. (Standard deviation is not presented due to the graph readability).

Since two different parameters (internal friction and tablet strength) were used to evaluate particle interactions at different pressures, the absolute values are not plotted in the graphs below (Figure 3 and Figure 4). Instead, the relative values, as a ratio of the value measured for the mixture to the value measured for pure microcrystalline cellulose *x/x*_Avicel PH102_, are applied to make a comparison feasible. Absolute values are provided as supplementary data.

During the measurements, relative humidity was recorded as moisture content of microcrystalline cellulose is known to be related to water content in the environment. All samples were analyzed under 30–45% relative humidity and 20–24 °C. After tablet production, each tablet was left at least 24 h under common laboratory conditions before analysis. This normal practice of time delay between tablet preparation and tablet tensile strength measurement was followed, even though both used components are not water soluble and not low-temperature melting, and time effects on tablets after their compression could be considered as negligible.

Under marginal and mild condition and pre-shear normal pressure of 9 kPa, two measurements were performed for each sample. Since a small number of independent observations was carried out, relative standard deviation (*RSD*) for this pressure was calculated using the equation *RSD* (%) = (|*x*_1 −_ *x*_2_| × *k*_n_ /*x*_mean_) × 100, where *k*_n_ is tabulated and equals 0.886 [63]. Obtained *RSD* ranged from 0.03% to 7.84%. In case of compaction condition, for each sample and experimental condition, five or six repetitions were performed. *RSD* for the measurements of tablet tensile strength was calculated using the same equation as in the case of marginal or mild stress conditions. However, the value of *k*_n_ equals 0.430 or 0.395 with regard to different number of repetitions. Obtained *RSDs* are listed in Table 3.

The *RSD* values for marginal or mild stress conditions are typically lower than that for compaction conditions. Unfortunately, this phenomenon cannot be avoided as it is likely caused by the different sample size used for analysis, which is inherent in the different test methods. Several milliliters of each sample were required in the case of marginal or mild stress condition in comparison with measurement under compaction condition and samples with volume lower than one milliliter. Because of that, a very small number of particles is involved in interparticle interaction analysis in the case of compaction condition and given values of tensile strength are more dependent on the composition of real samples that are used for each tablet preparation.

#### 3.3.1. Neusilin^®^ US2

The processes observed using SEM/BSE imaging are reflected by changing the level of particle interactions shown in Figure 3 including the measurement of the effective angle of internal friction and tablet strength. The figure shows increasing ratio, thus increasing particle interactions of Neusilin^®^ US2, as applied pressure increases. From the graph, it is also visible that the number of particle interactions is dependent on the present amount of Neusilin^®^ US2.

- Marginal or Mild Stress Condition

In case of no or mild pressure conditions (free flowing powder), increasing concentration of Neusilin^®^ US2 in the mixture leads to a decrease in the particle interactions. This observation indicates the ability of Neusilin^®^ US2 to improve flow properties and to work as a glidant. Comparing obtained results with those for a traditional surface-active glidant such as colloidal silica, there is no visible glidant effect maximum and negative over-loading effect observed in previous research oriented towards traditional glidants. It is probably given by different particle character leading to a different mechanism of action. It is known that traditional glidants with nanometer particle size improve flow properties by adhering to host particles and reducing contact points between them or filling cavities of the particles leading to their smoother surfaces. This abovementioned statement about different mechanisms of action can be confirmed by absolute values of the effective angle of internal friction (9 kPa pre-shear) obtained for the MCC binary mixture consisting of MAS (Neusilin^®^ US2, Neusilin^®^ S2) discussed in this study and identical mixtures containing colloidal silica (Aerosil^®^ 200) published in previous research by Tran et al. [52]. Values are listed in Table 4. The results indicate a correlation between increasing amount of presented MAS and the improvement of flow properties. A higher amount leads to a lower angle value and thus lower particle interactions without any maxima that is visible in case of Aerosil^®^ and reflects the necessity of ideal concentration assessment for a traditional glidant usage. Aerosil^®^-like behavior has been found for another MAS Neusilin^®^ UFL2 that is known for its glidant application [33]. Neusilin^®^ UFL2 is also powdered material composed of agglomerates of very fine particles that are adsorbed on host particles during mixing. Particulate characteristics of the same type and provided published findings, where the improvement of corn starch flow properties shows similar trend with visible effect maxima for both glidants, suggest most likely an identical mechanism of action distinctive for powdered glidants working on a surface-based principle. However, the granulated form of two studied MAS grades exhibits a different phenomenon and points out a different mechanism of action for this material type. Due to bigger particle size (approximately 100 µm) and low cohesion that is apparent from SEM/BSE observation, we assume Neusilin^®^ US2 and Neusilin^®^ S2 capability of flow property enhancement is largely based on volume effect and increasing minimum contact distance between host particles. Thus, their glidant activity is not assumed to be optimal-concentration-dependent and their application in pharmaceutical unit operations might be less sensitive to unexpected problems arising.

Analysis of variance, single-factor ANOVA test, was used to verify the statistically significant effect of type and concentrations of concerned glidants on the flowability of discussed powder systems. The significance of the difference was determined at 95% confident limit (*α* = 0.05) and considered to be significant at a level of *p* lower than a value obtained by application of Bonferroni correction setting the significance at *p* = *α*/*n*, where *n* equals number of measurement repetitions [64]. ANOVA data are enclosed as supplementary data of this article.

The ANOVA results indicate that the obtained *F* values are higher than *F_c_*_rit_ ones which means there is variance in studied systems. The hypothesis used for the ANOVA test of the “same mean values of all variances” is therefore rejected. The type and content of glidant play an important role in powder flow enhancement. The analysis revealed differences for mixtures containing higher concentrations of glidants, 5 and 10 percent. This indicates both the different influence of 3 compared glidants on the flowability of microcrystalline cellulose and the different mechanism of action of traditional glidants and MAS. However, two repetitions were done in the case of effective angle of internal friction measurement. Authors are aware of the fact it is not sufficient for correct and fully-fledged analysis for variance.

- Compaction Condition

In contrast to the free-flowing condition and low ratio values corresponding to reduction of particle interaction, the growth of ratios with subsequently increasing applied pressure reflects the creation of interaction between particles. Thus, for higher loaded mixtures, the presented results imply known suitability of studied silicate as carriers in solid drug delivery systems and ability to create sufficiently hard tablets. For instance in published literature, the influence of the silicate concentration on the tabletability of MCC (Vivapur^®^ 112)/Neusilin^®^ US2 and Neusilin^®^ UFL2 mixtures has been examined by the research group Gumaste et al. [27]. They observed only Neusilin^®^ US2 and Neusilin^®^ UFL2 could produce tablets with a tensile strength higher than 1 MPa among tested silicates. Another group, Hentzschel et al., also published that Neusilin^®^ US2 exhibits good tabletability [19]. From presented graphs, it can be deduced that the research group obtained for the studied MCC (Avicel^®^ PH102) mixture containing 50% Neusilin^®^ US2 slightly higher values of tensile strength compared to the mixtures prepared by our research group. Thus, the results of this investigation are in agreement with previous observations by other scientists.

In case of a mixture with 25% Neusilin^®^ US2, the ratio value is even higher than value of 1 and the particle interactions are stronger than that of the pure microcrystalline cellulose that is familiar for its good tableting properties. However, for lower content of Neusilin^®^ US2, a certain kind of saturation and a minor effect of pressures higher than 50 MPa on subsequent growth of particle interactions are visible. Another evident phenomenon is the influence of particle size of microcrystalline cellulose. Mixtures with a finer fraction evince less pronounced tablet strength. It is possible that the influence of Neusilin^®^ US2 is weakened due to the percolation of microcrystalline cellulose particles leading to a cellulose-like behavior. Next, in the case of applied pressure of 5 MPa, there is a noticeable difference between mixtures with 25% content of Neusilin^®^ US2 and other concentrations. They are characterized by a much higher tablet tensile strength. This is probably the result of Neusilin^®^ US2 particle fragility that could be considered as another bonding mechanism apart from plastic deformation. Due to high particle porosity, the surface area of MAS particles is primarily internal. It leads to low contact surface area and functional groups included in interparticle interactions, such as SiOH, that are normally poorly accessible. However, contact surface area rapidly increases after particle destruction and weak interparticle forces as Van der Waals forces as well. Moreover, functional groups become available. These obtained findings are in agreement with the findings that emerged from extensive research in a compaction field carried out by Nyström et al. and deals with issues of bonding and interparticle interactions [65,66,67,68,69]. They found that primary factors for the compactability of powders are bond mechanism and the surface area over which these bonds are active. Bonding surface area is obtained if the surface area of the particles in the tablet is large. This could be achieved just by the use of materials that undergo extensive fragmentation [69]. Pressure of 5 MPa is very low but sufficient to destroy the MAS particles. High concentration leads to the creation of large number of MAS particle interactions and the assumed reinforcement formulation is effective even under condition of such low pressure. However, a small number of Neusilin^®^ US2 particles is not enough to form strong reinforcement by their destruction, and do not give rise to such sufficient bonding in case of remaining mixtures.

#### 3.3.2. Neusilin^®^ S2

The results of the second representative of MAS, Neusilin^®^ S2, are provided in Figure 4. At low pressures, the behavior of the mixtures is similar to that of Neusilin^®^ US2, increasing concentration of Neusilin^®^ S2 results in a decrease in particle interactions that corresponds with an increase in its glidant effect. However, at higher pressures, the behavior of Neusilin^®^ S2 is slightly different. There is also its certain contribution to the formation of particle interactions, but it is not directly proportional to its content in mixture and the impact is not so intense. The most significant effect is visible for 5% concentration. The subsequent decrease in case of 10% is probably due to the fact Neusilin^®^ S2 itself produces weaker particle interactions than pure microcrystalline cellulose. This foundation is in agreement with the outcome of tablet tensile strength obtained for pure substances where Neusilin^®^ S2 exhibits lower values than microcrystalline cellulose. Therefore, its higher presence leads to a decrease in magnitude of particle interactions and the potential interconnections of Neusilin^®^ S2 particles that are formed tend to more likely play the role of defects in the structure of the prepared tablets. Values of tensile strength for pure excipients are presented in Table 5.

The ANOVA method was also applied to assess if attributes of materials have a statistically significant effect on their compaction. Similar to the analysis carried out for the effective angle of internal friction, obtained *F* values were higher than *F_c_*_rit_ ones and pointed to a dissimilar response of pure compounds to pressure application. The analysis detected that Neusilin^®^ US2 shows the most different compaction properties in comparison with other used substances.

From the results presented in Figure 3 and Figure 4, relation between the studied MAS behavior and applied pressure may be evaluated. The most obvious changes and differences in behavior reflecting possible creation of particle interactions occur at the applied compaction pressure of 5 MPa. It is in conformity with the fact that compaction pressures lower than 5 MPa did not result in tablet formation. At 5 MPa pressure, studied binary mixtures were tabletable. All prepared tablets were faultless without any visible cracks after the extraction from the die. However, tensile strength detected was lower than 1 MPa, which is typically desired for tablets to withstand stress during lifetime. Therefore, we believe that glidant function of MAS prevails applying pressures lower than 5 MPa. For MAS binder function, higher pressures are required. We also assume the observation trends at 5 MPa pressure are not the result of changing the way of interparticle interaction measurement, because the results at higher compaction pressures (above 5 MPa) follow in the results at minor or marginal pressures (below 5 MPa) and thus correspond with them logically.

## 4. Conclusions

**Figure 5:**
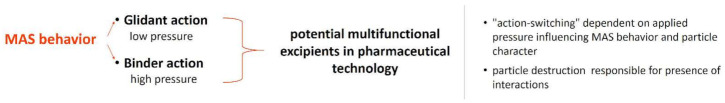
Graphical illustration of obtained findings.

This work was aimed at characterization of magnesium aluminometasilicates (MAS) behavior properties in binary mixtures with Avicel^®^ PH102 with respect to applied pressure and MAS concentration. It was found that both studied granulated MAS exhibited the “action-switching” behavior, reducing the inter-particle interactions at low pressures, while empowering them after reaching a certain threshold pressure, which is summarized graphically in Figure 5.

It was observed that the magnitude of the switching effect on particle interaction was given by the MAS ability to form stronger interactions compared to the mixture co-former, thus Neusilin^®^ US2 had more pronounced effect over Neusilin^®^ S2.

The results revealed the effect of applied pressure on the behavior and character of MAS particles is critical and it is responsible for the “action-switching” behavior. SEM/BSE images illustrated the ability of MAS particles to form interactions between one another and with particles of second excipient. These interactions develop only as a result of the original MAS particle destruction and reaching threshold pressure is necessary for this phenomenon to take place. A 5 MPa value of pressure was detected as the approximate threshold pressure value, but it is further dependent on total MAS loading as higher loading may facilitate the particle destruction at somewhat lower pressure. It is known that interparticle interactions are closely associated with particle contact surface area that increases with the concentration of MAS in mixtures. Thus, capacity to form interparticle interactions and its depletion are dependent on MAS concentration. It means that MAS work as a binder but with different contribution to final tablet strength with respect to their amount in mixtures.

In conclusion, this work revealed important application benefits of MAS usage in direct compression formulations. It was shown that MAS act as a glidant at low pressures because of their repulsive character but bonding ability increases after reaching the threshold pressure. MAS perform a binder activity after that. This foundation makes MAS a possible multifunctional excipient in the pharmaceutical technology field.

## Figures and Tables

**Table 1:** List of prepared mixtures.

Notation	Material (w/w%)				
	Microcrystalline Cellulose			MAS	
	100–150 µm	150–250 µm		Neusilin^®^ US2	Neusilin^®^ S2
1	99		-	1	-
2	95		-	5	-
3	90		-	10	-
4	75		-	25	-
5	-		99	1	-
6	-		95	5	-
7	-		90	10	-
8	-		75	25	-
9	99		-	-	1
10	95		-	-	5
11	90		-	-	10
12	-		99	-	1
13	-		95	-	5
14	-		90	-	10

**Table 2:** Physical characteristics of selected compounds.

Material	Particle Size Distribution			Particle Shape
	d_10_ (µm)	d_50_ (µm)	d_90_ (µm)	
100–150 µm MCC fraction	99.7	162.0	242.0	irregular
150–250 µm MCC fraction	132.0	229.0	341.0	irregular
Neusilin^®^ US2	46.1	108.0	206.6	spherical
Neusilin^®^ S2	64.2	140.1	249.3	spherical

**Table 3:** *RSD* values of tablet tensile strength with respect to applied compaction pressure.

**Applied Compaction Pressure (MPa)**	5	50	100	150
**Relative Standard Deviation *RSD* (%)**	5.35–23.97	1.25–12.06	2.32–9.67	1.68–10.45

**Table 4:** Values of effective angle of internal friction for pure compounds and prepared binary mixtures.

Glidant Concentration (w/w%)		Effective Angle of Internal Friction (°)				
		0	1	5	10	25
100–150 µm MCC fraction	Aerosil^®^ 200	40.38 ^a^	32.85 ^a^	36.49 ^a^	-	-
	Neusilin^®^ US2		33.28	31.33	30.05	24.55
	Neusilin^®^ S2		34.46	35.63	31.53	-
150–250 µm MCC fraction	Aerosil^®^ 200		31.66 ^a^	32.89 ^a^	-	-
	Neusilin^®^ US2		33.90	29.78	30.15	24.79
	Neusilin^®^ S2		33.63	33.57	32.14	-

^a^ adapted from Tran et al. [52].

**Table 5:** Tensile strength of tablets compacted from pure compounds.

Compound	Tensile Strength (MPa) - Applied Pressure 100 MPa	Tensile Strength (MPa) - Applied Pressure 150 MPa
100–150 µm MCC fraction	5.37 ± 0.30	7.07 ± 0.47
150–250 µm MCC fraction	5.35 ± 0.11	7.05 ± 0.27
Neusilin^®^ US2	7.72 ± 0.46	9.62 ± 0.38
Neusilin^®^ S2	4.12 ± 0.40	6.37 ± 0.11

## Data Availability

The data that support the findings of this study are contained within the article and associated supplementary data (www.mdpi.com/1996-1944/14/4/900/s1 Excel File).

## Supplementary Materials

The following are available online at https://www.mdpi.com/1996-1944/14/4/900/s1 Excel File.
